# Condylar Changes and Its Association with Age, TMD, and Dentition Status: A Cross-Sectional Study

**DOI:** 10.1155/2011/413639

**Published:** 2011-10-31

**Authors:** Anuna Laila Mathew, Amar A. Sholapurkar, Keerthilatha M. Pai

**Affiliations:** ^1^Department of Oral Medicine & Radiology, Marbasilios Dental College, Kothamangalam, Kerela 686691, India; ^2^Department of Oral Medicine and Radiology, College of Dentistry, King Khalid University, Gregor Abha 61471, Saudi Arabia; ^3^Department of Oral Medicine & Radiology, Manipal College of Dental Sciences, Manipal, Karnataka 576104, India

## Abstract

The present study was undertaken to evaluate the prevalence of radiographic changes in the condylar morphology and its association with age, clinical signs and symptoms of temporomandibular dysfunction and dentition status and also to evaluate the intra examiner and inter examiner reliability in assessing condylar changes using panoramic radiographs. A total of 75 subjects were recruited for the study. They were divided into 3 age groups. 20–40 yrs (Group A), 41–60 yrs (Group B) and 61 yrs and above (Group C). In each age group 25 subjects were evaluated both clinically and radiographically. The prevalence of radiographic changes in condylar morphology and symptoms of temporomandibular dysfunction was 81.3% and 18.6%, respectively. Radiographic abnormalities in the mandibular condylar morphology increased with age. They were seen more frequently in patients with clinical signs and symptoms of temporomandibular dysfunction and in patients with loss of teeth. Intra examiner and inter examiner reliability was high indicating a good reliability in assessing the condylar changes using panoramic radiograph.

## 1. Introduction

TMJ has many anatomic and functional features that make it unique and complex among the joints of the human body [1, 2]. Condylar remodeling is a physiologic process that aims to adapt the structure of the temporomandibular joint (TMJ) to meet the functional demands. It is based on an interaction between the mechanical forces sustained by the TMJ and the adaptative capacities of the condyle. The components of the TMJ are thought to retain their capacity for remodeling after growth has ceased and to continue to change their structure and morphology [3]. Correlations between morphological changes and age must be considered when evaluating panoramic radiographs.

Although structural changes are thought to be related to TMJ dysfunction, the mechanism of these structural changes affected by such reactive processes as remodeling, aging, and osteoarthrosis, still has not been completely clarified [4]. There have been some extensive examinations of the human condyle using autopsy specimens that have concentrated their attention on morphologic changes [5]. However, the changes in the human condyle with regard to aging or occlusal loss have not been completely elucidated. Temporomandibular disorders (TMD) constitute a complex set of specific entities with a wide range of reported prevalence. 

Panoramic radiography has been recommended as screening tool in patients with TMJ complaints and may be appropriate for determining gross bony changes in the condyle [6]. However, there can be a lack of correlation between radiographic findings and TMD symptomatology, and patients without TMD symptomatology can present with condylar changes demonstrated by panoramic imaging.

In addition, concerns persist about intraobserver and interobserver reliability in evaluating joint morphology using panoramic radiography [6]. Finally, there is controversy as to whether or not the number of remaining teeth affects condylar morphology despite evidence that morphological changes in elderly individuals are not associated (or only weakly associated) with TMD signs or dentition status.

The present study was undertaken to evaluate the prevalence of radiographic changes in the condylar morphology and its association with age, clinical signs and symptoms of temporomandibular dysfunction, and dentition status and also to evaluate the intraexaminer and interexaminer reliability in assessing condylar changes using panoramic radiographs.

## 2. Subjects and Methods

This study was carried out in our department which included patients who were advised for panoramic radiography for various purposes. A total of 75 subjects were recruited for the study. They were divided into 3 age groups: 20–40 yrs (Group A), 41–60 yrs (Group B), and 61 yrs and above (Group C). In each age group 25 subjects were evaluated both clinically and radiographically.

The following patients were excluded from the study:

when panoramic radiograph did not reveal the condylar anatomy clearly, patients with history of condylar fractures and surgery of condyles, those with developmental anomalies affecting the jaw or syndromes of the craniofacial structures, who had undergone orthodontic treatment, joint disorders like rheumatoid arthritis, gout, infective arthritis, history of parafunctional habits like bruxism, patients with limited mouth opening due to oral submucous fibrosis, space infections, or malignancy of the oral cavity.

Informed consent was taken from the patients prior to the history, clinical, and radiographic examination. A Questionnaire designed to record the symptoms was used. Clinical examination was performed to record signs of temporomandibular dysfunction.

### 2.1. Questionnaire Included the Following Questions

1. Is there any difficulty in mouth opening and whether it is experienced at any particular time of the day?
2. Is there any pain in front of the ear during opening and closing of the mouth?
3. Any pain in the muscles of the face during opening, yawning, chewing, speaking, swallowing and so forth?
4. Any sounds heard in the ear/front of ear during opening and closing of mouth?
5. History of pain in any other joint of the body?
6. History of clenching or grinding teeth?
7. Are you a denture wearer? Is the fit of the denture proper?

### 2.2. Clinical Examination

Patients underwent a detailed clinical examination. They were examined for mouth opening, deflection, deviation, lateral movements of the mandible, Muscles of mastication were palpiated for any tenderness. Temporomandibular joint was palpated, extraauricularly and intraauricularly for any tenderness. Temporomandibular sounds if any were recorded and confirmed with stethoscope.

Dentition was clinically examined. Dentition status was classified into 3 classes using Eichner Index. The molar and premolar contacts define the classification: Class A where patients had dental support in all four support zones, Class B where patients had dental support in one to three support zones, at least one contact in the molar or premolar area or contact in the front area only, and Class C where patients had no support zones at all, although a few teeth can still be remaining. Denture wearers were asked regarding the fit of the denture and its efficiency in chewing.

Panoramic images were taken using Planmeca PM 2002 CC machine in a standard manner. The films were processed in an automatic processor (Promax 5 L automatic X-ray film processor). Only high quality radiographs were considered for the study. Panoramic radiographs were examined by single observer under ideal viewing conditions. Radiographic changes in the condyle were recorded according to the definitions by Muir and Goss 1990 [7], Akerman et al. [8], and Flygare [9]. A normal condyle was given a score of 0, and an abnormal condyle was given a score of 1.

1. Flattening: loss of an even convexity or concavity of the joint out lines,
2. Osteophyte: local outgrowth of bone arising from a mineralized joint surface,
3. Erosion: local area of rarefaction in the cortical plate of a joint surface,
4. Sclerosis: thickening of the cortical bone on a joint surface,
5. Ely's cyst (sub cortical cyst): Rounded radiolucent area that may be just below the cortical plate or deep in trabecular bone.

In order to assess the intraobserver and interobserver reliability 10 radiographs in each age group (a total of 30 radiographs) were selected at random and reexamined after a time gap of 3 months by the same observer and another observer trained in interpreting panoramic images.

## 3. Statistical Analysis

Data were entered into the computer and analyzed using SPSS software version 15. A normal condyle was given a score of 0, and abnormal condyle was given a score of 1. Chi square for trend was used to assess whether the number of subjects and number of condyles with radiographic changes in the condylar morphology increases with advancing age. Chi square test was used to analyze the association between radiographic changes in the condylar morphology and clinical signs and symptoms of temporomandibular dysfunction. Kappa statistics for agreement was used to assess intraexaminer and interexaminer reliability. Kappa statistics were interpreted as <0 = poor agreement, 0.00–0.20 = slight agreement, 0.21–0.40 = fair agreement, 0.41–0.60 = moderate agreement, 0.61–0.80 = substantial agreement, 0.81–0.99 = almost perfect agreement, and 1.00 = perfect agreement. *P* value equal to or less than 0.05 was considered statistically significant. Fisher's exact test was used for assessing the association between radiographic changes in condylar morphology and dentition status.

## 4. Results

Out of the total 75 patients who were examined radiographically 61 (81.3%) had radiographic changes in condylar morphology. The prevalence of radiographic changes in the condylar morphology was found to be relatively lower in Group A (Table 1). As the age increased, there was a statistically significant increase in the number of subjects with radiographic changes in condylar morphology (*P* value 0.012). 

The number of condyles affected in each group is shown in Table 2. The Group B an C showed more radiographic abnormalities as compared to Group A. As the age increased there was a statistically significant increase in the number of condyles affected (*P* value 0.009). 

Table 3 shows the radiographic changes in the condylar morphology in three age groups. In Group A, 9 subjects had normal condyles, in Group B, 3 subjects had normal condyles, and in Group C, 2 subjects had normal condyles. Abnormal condyles on the right side were observed in 15 subjects in the Group A, 17 subjects in Group B, and 18 subjects in Group C respectively. Abnormal condyles on the left side were observed in 16 subjects in Group A, 20 subjects in Group B, and 19 subjects in Group C respectively. Abnormality in both condyles was observed in 12 subjects in Group A, 16 subjects in Group B and 15 in Group C, respectively. Abnormalities in one or both condyles were observed in 16 subjects in Group A, 22 subjects in Group B, and 23 subjects in Group C.

Table 4 shows the distribution of various radiographic condylar changes among the three age groups. Flattening was observed in 60 subjects (80%). It was the most common finding, followed by osteophyte (16%), sclerosis (12%), erosion (8%), and Ely's cyst (6.7%). Flattening, erosion, and sclerosis were observed more in the older age group as compared to the younger age group. Osteophyte and Ely's cyst showed a lower prevalence as the age advanced.

Table 5 shows the correlation between reported symptoms of TMJ dysfunction and radiographic changes in condylar morphology. Out of the 75 subjects a total of 14 subjects reported of symptoms suggestive of TMJ dysfunction. 1 patient reported of difficulty in mouth opening, 11 patients reported of pain during jaw movements, and 3 reported of clicking sounds. These reported symptoms were more prevalent in patients who had radiographically abnormal condylar morphology. Difficulty in mouth opening was reported only in one subject with abnormal condylar morphology radiographically, and there was no statistically significant correlation between reported difficulty in mouth opening and radiographic abnormalities in the condylar morphology (*P* value 1.00). Though pain while opening and closing the mouth, yawning, and chewing was more frequent in subjects with abnormal condyles (10 subjects) than those with normal condyles (1 subject), there was no statistically significant association between radiographic abnormalities in the condylar morphology and these symptoms (*P* value 0.273). TMJ sound (clicking) was observed more in subjects with radiographically normal condyles (2 subjects) as compared to subjects with abnormal condyles (1 subject), and there was no statistically significant association between the abnormal radiographic condylar findings and TMJ sounds (*P* value 0.088). 

Table 6 shows the correlation between clinical signs and radiographic changes in condylar morphology. Although one subject reported of reduced mouth opening, there was no statistically significant association between reduced mouth opening and radiographic abnormalities in condylar morphology. Limited lateral movement was demonstrated in 3 subjects, and all of them had radiographic abnormalities in condylar morphology, but there was no statistically significant association between limited lateral movement and radiographic abnormalities in condylar morphology (*P* value = 1.00). Deviation of the mandible was observed in 8 subjects, out of whom 3 had normal condyles radiographically. Though deviation of mandible was observed more frequently in subjects with abnormal condylar morphology radiographically, it did not reach any statistical significance (*P* value = 0.164). Deflection of mandible was observed in 2 subjects, and both had radiographic abnormalities in condylar morphology, but there was no statistically significant association between deflection of condyle and radiographic abnormalities in condylar morphology (*P* value = 1.00). TMJ sound (click) was present in 8 subjects where abnormal condylar morphology was seen radiographically in 6 subjects; however there was no statistically significant association between TMJ sounds and radiographic abnormalities in condylar morphology (*P* value = 0.638).

Tenderness of TMJ was observed in 6 subjects, and all of them had abnormal condyles radiographically, but it did not reach statistical significance (*P* value = 0.586). Tenderness of muscles of mastication was observed only in 1 subject who had abnormal radiographic condylar morphology, but there was no statistical significant association between tenderness of muscles of mastication and radiographic abnormalities in condylar morphology (*P* value 1.00). 

Table 7 shows the association between radiographic changes in condylar morphology and dentition status. The frequency of abnormal radiographic condylar changes was less in Group A in comparison with Group B and Group C. However the association was not statistically significant (*P* value 0.081).

Table 8 shows the intraexaminer reliability for various radiographic findings. The intra-class coefficient values showed high reliability for all the radiographic condylar findings. The intra-class coefficient was 0.92 = almost perfect agreement for flattening and osteophyte and 1.00 = perfect agreement for erosion, sclerosis, and Ely's cyst. 

Table 9 shows the interexaminer reliability for various radiographic findings. The inter-class coefficient values showed high reliability for all the radiographic condylar findings. The inter-class coefficient was 0.84 = almost perfect agreement for flattening, 0.94 = almost perfect agreement for osteophyte, 0.90 = almost perfect agreement for erosion and sclerosis, and 1.00 = perfect agreement for Ely's cyst.

## 5. Discussion

The prevalence of changes in condylar morphology was more in individuals above 40 yrs (90%) as compared to those below the age of 40 (64%) which was statistically significant (*P* value 0.012). It was also observed that, as the age increased, the number of condyles affected also increased. The Group B and Group C had more condylar changes as compared to Group A, which was statistically significant (*P* value 0.009). This observation is in agreement with the observations of Muir and Goss [7], Huumonen et al. [10], and Takayama et al. [11] that absence of morphologic variation was much more common in the younger age group and age is a factor that determines the degree of remodeling, though there is no direct linear relationship. Because the adaptive or degenerative changes in the temporomandibular joints appear over a long period of time, it is understandable that the condylar changes increase with advancing age.

However the findings of our study were not in accordance with the observations of a few studies which showed condylar changes to be more prevalent in younger age groups or showed condylar changes in all age groups [12]. Crow et al. [6] observed morphologic condylar changes in panoramic radiographs in all adult age ranges. They attributed the high prevalence of minor condylar changes seen in both the patients with TMD and general dental population to remodeling.

 In our study subjects with abnormal condyles on one or both sides showed more number of abnormal findings in Group C and more on the left side which is in agreement with the observations by Takayama et al. [11]. This may be an incidental observation.

Flattening was observed in 60 subjects (80%) and this was the most common finding, followed by osteophyte (16%), sclerosis (12%), erosion (8%), and Ely's cyst (6.7%). Flattening, erosion, and sclerosis were observed more in older age group. Osteophyte and Ely's cyst showed a lower prevalence as the age increased. This finding is in agreement with the findings of Sato et al. [13], Hiltunen et al. [14], and Takayama et al. [11]. In their studies, the most frequent radiographic finding was flattening followed by erosion, osteophyte, and sclerosis.

Out of the 75 subjects, 14 subjects had symptoms of TMD (18.6%). The symptoms were in the form of difficulty in mouth opening, pain on jaw movements, and TMJ sounds. The prevalence of reported symptoms in our study is comparable to the observations by De Kanter et al. [15], Celic et al. [16], Farsi [17], and Bonjardim et al. [18]. It is less prevalent as compared to the observations by Locker and Slade [19], Otuyemi et al. [20], and Feteih [21], and the prevalence is higher than reported in studies by Goulet et al. [22] and Gesch et al. [23].

The diversity among the prevalence of TMD among different studies can be attributed to the differences in the age groups, the sample sizes, their composition (TMD and non-TMD patients), and the number of examiners, as well as the diagnostic criteria used.

Though there was no statistically significant association between reported symptoms and radiographic changes in condylar morphology, the symptoms were more frequent in those with radiographically abnormal condyles. Clicking was the only TMJ sound that was observed, and this was more in subjects with radiographically normal condyles as compared to those with radiographically abnormal condyles. 

It was also observed that there was no statistically significant association between clinical signs and radiographic changes in condylar morphology. Though there was no statistically significant correlation between limited lateral movement and abnormal condylar findings it was observed more frequently in those with radiographic abnormal condyles.

There was no statistically significant association between radiographic changes in the condylar morphology and clinical signs and symptoms temporomandibular dysfunction, and this was in accordance with a few other studies by Sato et al. [13], Hiltunen et al. [24], Crow et al. [6], Hansson et al. [25], Bush et al. [26], and Huumonen et al. [10]. However this was not in agreement with the results of the studies by Flygare et al. [9], Takayama et al. [11] where an increased frequency of radiographic morphologic changes in TMJ was noted in patients with pain when compared with patients without symptoms. This increase in frequency may be because subjects in their studies had symptomatic TMJs.

The association between changes in the mandibular condyle and dentition status has been widely studied. In our study it was observed that subjects in Group A had fewer abnormal condyles as compared to Group B or Group C. However the difference was not statistically significant. This observation is in agreement with the previous studies by Pereira et al. [5], Sato et al. [13], Hiltunen et al. [14], Crow et al. [6], and Takayama et al. [11], but not in agreement with Muir and Goss [7], Giesen et al. [27], and Harriman et al. [28] that showed an association between dentition status and condylar changes. 

The intra-class and inter-class coefficient, were close to 1, and there was almost perfect agreement. This is in agreement with the observations made by Crow et al. [6], and not in agreement with the observation by Vidra et al. [29], where the observer consistency in radiographic assessment of condyles using panoramic views was mediocre or poor for surface and shape of the condyle.

## 6. Conclusion

Following were the conclusions drawn from our study. The prevalence of radiographic changes in condylar morphology was 81.3%, and the prevalence of symptoms of TMD was 18.6%. Radiographic abnormalities in the condylar morphology increased with age. They were seen more frequently in patients with clinical signs and symptoms of TMD and in patients with loss of teeth, although they did not reach statistical significance. Intraexaminer and interexaminer reliability was high indicating a good reliability in assessing the condylar changes using panoramic radiograph, the most commonly used screening radiograph.

Radiographic appearance of TMJ varied widely, remodeling changes were commonly seen, and there was no direct linear relationship between age and radiographic changes in condylar morphology. Since there is no statistically significant association between radiographic changes in condylar morphology and clinical signs and symptoms and dentition status, caution should be exercised not to over-estimate the significance of radiologic abnormality. Minor changes in the radiographic image of the condyle of the patients with TMD may have no relevance and should not be used to infer a diagnosis. Small sample size and usage of only panoramic radiograph to assess the condylar changes were the limitations. So further studies with a larger sample size and other radiographic modalities for studying condylar morphology are recommended.

## Figures and Tables

**Table 1:** Prevalence of radiographic condylar changes according to age.

Age group	Number of patients with condylar changes
20–40 (Group A)	16 (64%)
41–60 (Group B)	22 (88%)
61 and above (Group C)	23 (92%)

(*P* value 0.012) Chi square for trend.

**Table 2:** Number of condyles affected in each age group.

Age group	Number of condyles affected
Group A	28 (56%)
Group B	38 (76%)
Group C	40 (80%)

(*P* value 0.009) Chi square for trend.

**Table 3:** Radiographic changes in condyles in three age groups.

Condyles	Group A	Group B	Group C	Total
Normal in both	9	3	2	14 (18.7%)
Abnormal in right	15	17	18	50 (66.6%)
Abnormal in left	16	20	19	55 (73.3%)
Abnormal in both	12	16	17	45 (60%)
Abnormal in one or both	16	22	23	61 (81.3%)

**Table 4:** Distribution of various radiographic findings among three age groups.

Radiographic findings	Group A	Group B	Group C	Total (%)
Flattening	16	21	23	60 (80%)
Osteophyte	6	5	1	12 (16%)
Erosion	1	2	3	6 (8%)
Sclerosis	3	1	5	9 (12%)
Ely's cyst	1	4	0	5 (6.7%)

**Table 5:** Correlation between symptoms and radiographic changes in condyles.

Symptom	Number of patients with reported symptoms	Abnormal condyle	Normal condyle	Percentage of patients with reported symptoms	*P* value
Difficulty in mouth opening	1	1	0	(1.3%)	1.00
Pain while opening, closing, yawning and chewing	11	10	1	(13.6%)	0.273
TMJ sounds(Clicking)	3	1	2	(4%)	0.088

**Table 6:** Correlation between clinical signs and radiographic changes in condyles.

Signs	Number of patients and percentage	Normal condyle	Abnormal condyle	*P* value
Reduced interincisal opening	0	0	0	—
Limited lateral movement	3 (4%)	0	3	1.00
Deviation of mandible	8 (10.6%)	3	5	0.164
Deflection of mandible	2 (2.62%)	0	2	1.00
TMJ sounds (Click)	8 (10.6%)	2	6	0.638
Tenderness of TMJ	6 (8%)	0	6	0.586
Tenderness of muscles	1 (1.3%)	0	1	1.00

**Table 7:** Association between radiographic changes in condyles and dentition status.

Dentition		Radiographic finding		Total
		Normal	Abnormal	
A	Count	13	38	51
	% within dentition	25.5%	74.5%	100.0%
B	Count	1	17	18
	% within dentition	5.6%	94.4%	100.0%
C	Count	0	6	6
	% within dentition	0%	100.0%	100.0%
Total	Count	14	61	75
	% within dentition	18.7%	81.3%	100.0%

(*P* value 0.081), not statistically significant.

**Table 8:** Intraexaminer reliability.

Radiographic finding	Intraclass coefficients
Flattening	0.92
Osteophyte	0.92
Erosion	1
Sclerosis	1
Ely's cyst	1

**Table 9:** Inter examiner reliability.

Radiographic finding	Inter-class coefficients
Flattening	0.842
Osteophyte	0.942
Erosion	0.90
Sclerosis	0.90
Ely's cyst	1
